# Neutrophils and lymphopenia, an unknown axis in severe COVID-19 disease

**DOI:** 10.1371/journal.ppat.1009850

**Published:** 2021-09-02

**Authors:** Hernán F. Peñaloza, Janet S. Lee, Prabir Ray

**Affiliations:** 1 Acute Lung Injury Center of Excellence, Division of Pulmonary, Allergy, and Critical Care Medicine, Department of Medicine, University of Pittsburgh, Pittsburgh, Pennsylvania, United States of America; 2 Department of Immunology, University of Pittsburgh School of Medicine, Pittsburgh, Pennsylvania, United States of America; University of Colorado Denver, UNITED STATES

## Abstract

The Coronavirus Disease 2019 (COVID-19) is caused by the betacoronavirus Severe Acute Respiratory Syndrome Coronavirus 2 (SARS-CoV-2) virus that can mediate asymptomatic or fatal infections characterized by pneumonia, acute respiratory distress syndrome (ARDS), and multi-organ failure. Several studies have highlighted the importance of B and T lymphocytes, given that neutralizing antibodies and T cell responses are required for an effective immunity. In addition, other reports have described myeloid cells such as macrophages and monocytes play a major role in the immunity against SARS-CoV-2 as well as dysregulated pro-inflammatory signature that characterizes severe COVID-19. During COVID-19, neutrophils have been defined as a heterogeneous group of cells, functionally linked to severe inflammation and thrombosis triggered by degranulation and NETosis, but also to suppressive phenotypes. The physiological role of suppressive neutrophils during COVID-19 and their implications in severe disease have been poorly studied and is not well understood. Here, we discuss the current evidence regarding the role of neutrophils with suppressive properties such as granulocytic myeloid-derived suppressor cells (G-MDSCs) and their possible role in suppressing CD4^+^ and CD8^+^ T lymphocytes expansion and giving rise to lymphopenia in severe COVID-19 infection.

The Coronavirus Disease 2019 (COVID-19) is a heterogeneous disease that ranges from asymptomatic [1] to severe disease with fatal outcome [2,3]. Severe COVID-19, initiated by successful viral infection and replication within type I and II alveolar epithelial cells [4,5], is characterized by excessive lung inflammation and injury and caused by a delayed interferon response followed by lymphopenia and cytokine storm that together impair local viral clearance and injury resolution [6–8]. The elderly, which commonly present with lymphopenia, is the most affected group by severe COVID-19 and registers the highest mortality rate [3]. Importantly, the mechanisms behind lymphopenia during severe COVID-19 remain unknown. Despite the advent of vaccines that have shown high efficacy in preventing severe disease and in providing protection from infection [9,10], the slow vaccination rate in developing countries and the emergence of variants of concern [11,12] raise the importance of understanding the mechanisms behind the dysregulated immune response that leads to severe COVID-19 in some individuals.

An integrated analyses of the immune response obtained from the bronchoalveolar lavage (BAL) samples of patients affected by COVID-19 suggest that the early viral infection of alveolar macrophages and monocyte-derived macrophages by Severe Acute Respiratory Syndrome Coronavirus 2 (SARS-CoV-2) triggers the production of T lymphocyte chemokines such as CCL24, CCL7, and CCL8 that promote the recruitment of T lymphocytes to the lungs [13]. Further production of interferon gamma (IFN-γ) by recruited T cells activates macrophages and induces the production of cytokines required for T lymphocyte activation, such as CXCL10, CCL4, and IL-1β [13]. This early circuit of monocytes and T lymphocytes is replaced later by a robust recruitment of neutrophils comprising around 50% of the total cells found in BAL [13].

Several studies have demonstrated that during an acute viral pulmonary infection in humans and animal models, neutrophils are actively recruited to the site of infection, amplifying lung inflammation without contributing to viral clearance [14]. In fact, a recent report shows that enriched neutrophils in the nasal mucosa in humans and mice lead to severe symptomatology during respiratory syncytial virus infection [15].

Initial analyses of neutrophil function from endotracheal aspirates during COVID-19 showed that neutrophils can also harbor viral particles intracellularly, exhibiting a pro-inflammatory phenotype, characterized by the production of IL-6 and tissue factor [16]. Other studies have shown that during COVID-19, neutrophils cause tissue damage, thrombosis, and blood clots through degranulation—or release of protein content stored in granules—and NETosis—extrusion of DNA and chromatin into the extracellular space [17–19]. These data indicate that during COVID-19, neutrophils actively contribute to inflammation, immunopathology, and severe disease. Neutrophils are myeloid granulocytic cells derived from early committed granulocyte–monocyte progenitors (GMPs) within the bone marrow [20], which are rapidly expanded during sepsis or infection [21]. Despite the fact that neutrophils are typically described as pro-inflammatory cells, a recent work shows that neutrophils are heterogeneous and remarkably plastic cells that display not only pro-inflammatory phenotypes, but also suppressive functions [22]. Specifically, studies using mouse models have shown that during viral, bacterial, and parasitic infections, neutrophils may acquire suppressive phenotypes characterized by inhibition of T lymphocyte proliferation and activity through arginase-1, inducible nitric oxide synthase (iNOS), and reactive oxygen species (ROS) production and induction of T cell anergy through PD-L1/PD-1-mediated interaction [22–32]. This suppressive neutrophil subset, referred to as granulocytic myeloid-derived suppressor cells (G-MDSCs), has also been identified in humans [23,26,30–32].

During an inflammatory response induced by infection, the local production of cytokines such as G-CSF, GM-CSF, and M-CSF triggers granulopoiesis of neutrophils and monocytes that are recruited to the site of infection [30–32]. Cytokines produced in the local microenvironment subsequently dictate the function of these cells, helping to clear the infection and resolve inflammation [30–32]. However, during pathologic states of dysregulated or unresolved inflammation, aberrant activation of recruited neutrophils and monocytes may occur in the local environment, characterized by impaired phagocytic capability with increased production of anti-inflammatory cytokines such as IL-10 and transforming growth factor beta (TGF-β), increased production of ROS, and higher expression of iNOS, arginase-1, and PGE2 that altogether suppress the activity and proliferation of CD4^+^ and CD8^+^ T cells and promote the expansion of regulatory T (Treg) cells [30–32].

Recently, the role of monocytic MDSCs in COVID-19 disease outcome has been described [33]. In this manuscript, we have primarily focused on the contribution of G-MDSCs, which are derived from the neutrophil GMP [30].

Several studies have described the expansion of suppressive neutrophils or G-MDSCs during COVID-19 and the association of these cells to disease severity [34–38]. Studies have shown that during COVID-19, neutrophils are a heterogeneous group of cells comprised of both immature and mature cells with pro-inflammatory and suppressive properties [34,35]. A population of immature CD10^neg^ neutrophils that exhibit a suppressive phenotype was shown to produce high levels of Ca2^+^ binding proteins S100A8/A9 [34] that are typically produced by G-MDSCs in human and mice and promote further expansion and accumulation of G-MDSCs [39–41]. Interestingly, the presence of G-MDSCs as well as the production of S100A8/A9 were overrepresented in patients with severe COVID-19 compared to patients with mild disease or healthy controls [34]. Still, another study showed that, during COVID-19, immature and mature subsets of neutrophils identified in peripheral blood express not only classical pro-inflammatory markers such as neutrophil elastase and myeloperoxidase, but also suppressive markers typically expressed by G-MDSCs such as arginase-1 and PD-L1 [35,42]. Immature PD-L1^+^ neutrophils were significantly expanded after 10 days post-symptom onset and were overrepresented in severe COVID-19 patients compared with patients with mild COVID-19 [39].

Further studies provided evidence that the numbers of suppressive immature neutrophils and/or G-MDSCs expanded during severe COVID-19 infection are associated with lymphopenia and disease severity [36–38]. Whereas 2 studies determined that the numbers of suppressive CD16^+^LOX1^+^ G-MDSCs in circulation were increased during severe COVID-19 and constituted a strong predictor of disease severity [36,37], a third study showed that the expansion of CD10^neg^ immature neutrophils is associated with decreased numbers of CD4^+^ T cells, CD8^+^ T cells, VD1 T cells, and VD2 T cells in circulation, increased cytokine production, and disease severity [38]. Collectively, these studies strongly suggest that the expansion and activity of G-MDSCs are associated with T lymphocyte numbers and disease severity.

Recent works that identified the presence of G-MDSCs (HLA-DR^neg^Lin^neg^CD33^+^CD11b^+^CD15^+^CD14^neg^) by flow cytometry in the peripheral blood of COVID-19 patients provided mechanistic insights by which these cells can impair T cell activity and cause lymphopenia [43,44]. Ex vivo studies determined that G-MDSCs isolated from COVID-19 patients were able to suppress the proliferation of T lymphocytes previously stimulated with *Staphylococcus* enterotoxin-B [43,44] and inhibited the production of IFN-γ by CD3^+^ T lymphocytes stimulated with SARS-CoV-2 Spike and Nucleocapsid-derived peptides [43]. Mechanistically, the inhibition of T lymphocyte function by G-MDSCs obtained from COVID-19 patients has been shown to be dependent upon iNOS and TGF-β, as the treatment with a neutralizing antibody against TGF-β or with the NOS2 inhibitor, L-N^G^-nitro arginine methyl ester (L-NAME), reestablished the production of IFN-γ by T lymphocytes ex vivo [43]. Consistent with previous studies discussed [34–36,38], patients admitted to the intensive care unit (ICU) due to severe COVID-19 had increased proportions of G-MDSCs in the peripheral blood compared to non-ICU patients with milder disease, supporting the hypothesis that these cells may be potentially involved in COVID-19 severity [43]. Moreover, similar analyses showed that the proportion of G-MDSCs in circulation from nonsurvivors was significantly higher compared to that in survivors [43].

IL-10 is one important mechanism used by MDSCs to modulate inflammation [24,30,31,45–47]. In mice, the production of IL-10 by MDSCs has been described in acute lung bacterial infection [45–49]. During COVID-19, IL-10 production has been identified as a strong predictor of disease severity as IL-10 is elevated in patients with severe disease compared to nonsevere disease [50]. It remains unknown whether G-MDSCs produce IL-10 during COVID-19 and whether the increased production of IL-10 observed during severe disease is a cause or a consequence. Nevertheless, the positive association between G-MDSC expansion and IL-10 production with disease severity raises the possibility that G-MDSCs may be one source of IL-10.

Several hypotheses to explain lymphopenia during severe COVID-19 have been proposed, including cytokine storm, T lymphocyte exhaustion, T lymphocyte infection by SARS-CoV-2, and interference of T cell activation by SARS-CoV-2 [51]. None of these hypotheses have been fully evaluated and necessitate a deeper understanding of the inflammatory and suppressive role of neutrophils during COVID-19 in local and multisystemic inflammation, T cell suppression, and injury resolution.

The data discussed in this manuscript suggest that the expansion and activation of G-MDSCs during COVID-19 may be involved in lymphopenia and severe disease. The suppressive function of G-MDSCs include (I) impaired T cell proliferation and suppressed IFN-γ cytokine production; (II) anergy of effector CD8^+^ cytotoxic T cells and CD4^+^ helper T cells; or (III) expansion of Treg cells [30,31]. Ex vivo evidence shows that G-MDSCs isolated from COVID-19 patients inhibited the proliferation of T lymphocytes and suppressed their ability to produce IFN-γ [43,44]. Interestingly, patients with severe COVID-19 present an increased proportion of PD-1^+^CD8^+^ and PD-1^+^CD4^+^ T lymphocytes compared to patients with mild disease [52,53]. Given that G-MDSCs can induce T cell anergy through PD-L1–PD-1 interaction [31] and that PD-L1^+^ neutrophils are expanded during COVID-19 [35], it raises the possibility that PD-L1^+^G-MDSCs can induce T cell anergy through the interaction of PD-L1–PD1. Finally, studies have shown that Tregs are not significantly expanded during COVID-19, and patients with severe COVID-19 exhibit a reduction rather than increase in CD45RA^+^ Treg cells compared to patients with mild disease [53,54]. This suggests that increased Treg expansion does not occur in severe disease and therefore is not a mechanism by which G-MDSCs may predispose to severe COVID-19.

Ex vivo studies analyzing the properties of human G-MDSCs in circulation have demonstrated similarities between human and mouse G-MDSCs to inhibit T cell responses [25–27,29]. These data indicate that the role of G-MDSCs initially identified in mouse models could be potentially extrapolated to human diseases, inviting the possibility to study the effect and mechanisms of G-MDSCs in lymphopenia using humanized mouse models [55]. In fact, a recent study identified the presence of immature neutrophils in the lungs, blood, and the bone marrow of humanized mice infected with SARS-CoV-2 [56]. This study also provided mechanistic insights regarding the molecules involved in the expansion of immature neutrophils such as the alarmins S100A8/A9 that were up-regulated during SARS-CoV-2 infection, and in which inhibition through Paquinimod, a small molecule inhibitor of S100A9, resulted in reduced lung inflammation and disease severity following viral infection [56]. Interestingly, S100A8/A9 was massively produced by suppressive immature neutrophils in patients with severe COVID-19 [34]. Targeting S100A8/A9 in patients could potentially limit the accumulation of suppressive neutrophils/G-MDSCs and improve disease prognosis.

An important question that remains unsolved is whether the temporality of G-MDSCs expansion affects disease outcome. It is possible that, during severe COVID-19, the early expansion of G-MDSCs may impair the activity and proliferation of T lymphocytes required for viral clearance. In contrast, the expansion of G-MDSCs occurring at later time points may improve lung injury resolution resulting in milder COVID-19. Overall, the data thus far available invite the possibility that G-MDSCs may be an important contributor of impaired T lymphocyte function and lymphopenia observed in severe COVID-19 (Fig 1). Future studies in humans and humanized mouse models focusing on the interaction between G-MDSCs and T cells will increase understanding of the role of neutrophils during COVID-19. This research area will provide the initial steps forward in designing innovative therapeutic agents useful to prevent severe disease, facilitating patient management and treatment during mild and severe COVID-19.

**Fig 1:**
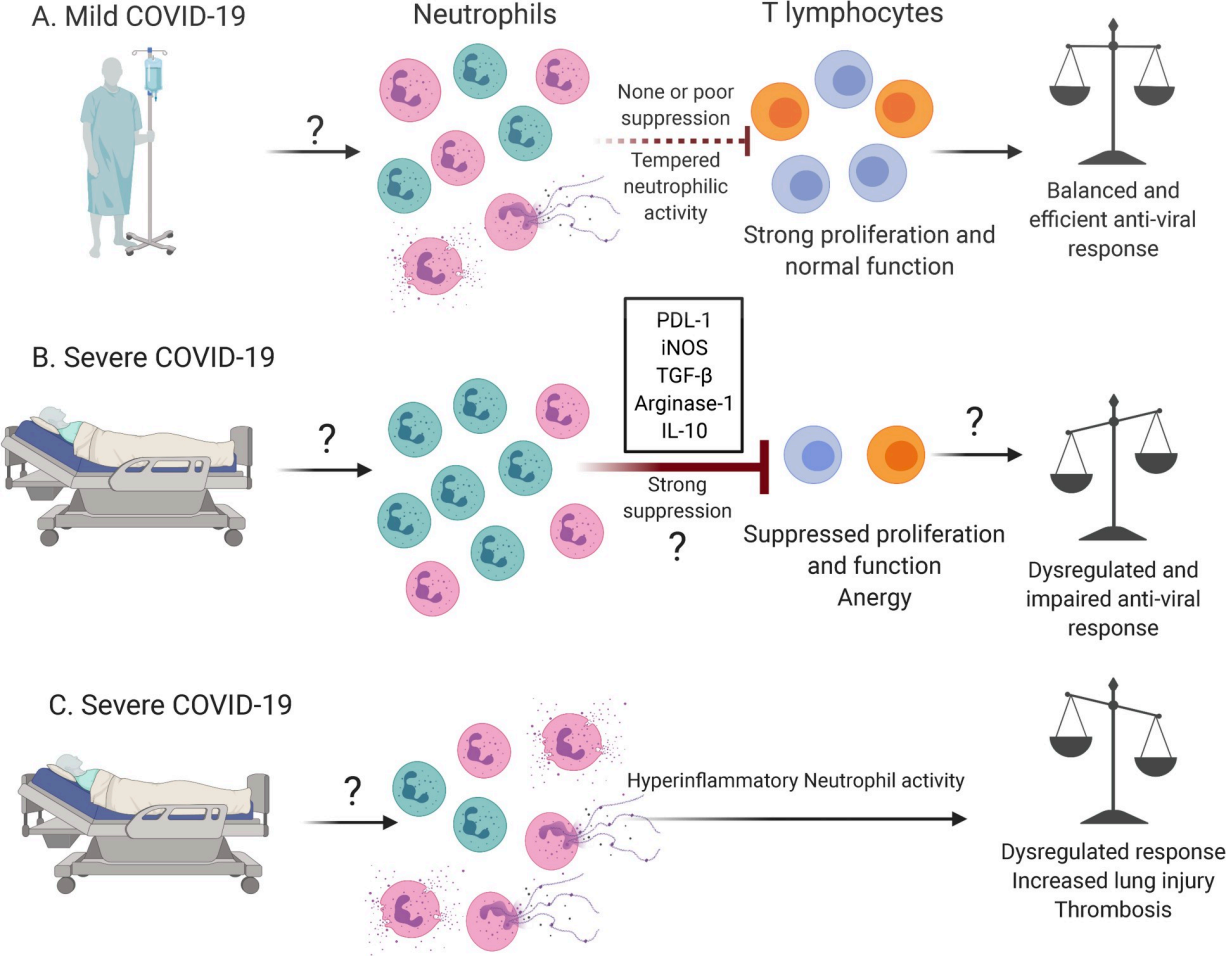
Neutrophils and G-MDSCs during COVID-19. Based on the current evidence, we hypothesize that the limited expansion of G-MDSCs (green) compared with classical pro-inflammatory neutrophils (pink) during (A) mild COVID-19 would be beneficial for the development of a balanced antiviral response due to robust proliferation of CD4^+^ (blue) and CD8^+^ (orange) T lymphocytes. In contrast, during (B) severe COVID-19, we speculate that the enhanced expansion of suppressive neutrophils may strongly suppress T cell proliferation and function, dysregulating the antiviral immune response and promoting excessive organ injury and diminished survival. The factors that promote the expansion of suppressive neutrophils as well as the putative suppressive mechanisms employed by these cells still need to be elucidated, and, if confirmed, it would add a new perspective of how neutrophil activity is involved in severe COVID-19 not limited to the classical view in which (C) pro-inflammatory neutrophils contribute to injury and disease through degranulation, NETosis, and pro-inflammatory cytokine production. The figure presented in this manuscript was generated with BioRender.com. COVID-19, Coronavirus Disease 2019; G-MDSC, granulocytic myeloid-derived suppressor cell; IL-10, interleukin 10; iNOS, inducible nitric oxide synthase; PDL-1, programmed death-ligand 1; TGF-β, transforming growth factor beta.
